# Comparison of three small-area mortality metrics according to urbanity in Korea: the standardized mortality ratio, comparative mortality figure, and life expectancy

**DOI:** 10.1186/s12963-020-00210-7

**Published:** 2020-07-03

**Authors:** Ikhan Kim, Hwa-Kyung Lim, Hee-Yeon Kang, Young-Ho Khang

**Affiliations:** 1 Department of Health Policy and Management, Seoul National University College of Medicine, Seoul, South Korea; 2 Department of Health Policy and Management, Jeju National University College of Medicine and Graduate School of Medicine, Jeju, South Korea; 3 Institue of Health Policy and Management, Seoul National University Medical Research Center, Seoul, South Korea

**Keywords:** Age distribution, Bias, Health status, Life expectancy, Mortality, Republic of Korea

## Abstract

**Background:**

This study aimed to compare three small-area level mortality metrics according to urbanity in Korea: the standardized mortality ratio (SMR), comparative mortality figure (CMF), and life expectancy (LE) by urbanity.

**Methods:**

We utilized the National Health Information Database to obtain annual small-area level age-specific numbers of population and deaths in Korea between 2013 and 2017. First, differences in the SMR by urbanity were examined, assuming the same age-specific mortality rates in all small areas. Second, we explored the differences in ranking obtained using the three metrics (SMR, CMF, and LE). Third, the ratio of CMF to SMR by population was analyzed according to urbanity.

**Results:**

We found that the age-specific population distributions in urbanized areas were similar, but rural areas had a relatively old population structure. The age-specific mortality ratio also differed by urbanity. Assuming the same rate of age-specific mortality across all small areas, we found that comparable median values in all areas. However, areas with a high SMR showed a strong predominance of metropolitan areas. The ranking by SMR differed markedly from the rankings by CMF and LE, especially in areas of high mortality, while the latter two metrics did not differ notably. The ratio of CMF to SMR showed larger variations in small areas in rural areas, particularly in those with small populations, than in metropolitan and urban areas.

**Conclusions:**

In a comparison of multiple SMRs, bias could exist if the study areas have large differences in population structure. The use of CMF or LE should be considered for comparisons if it is possible to acquire age-specific mortality data for each small area.

## Background

Representative metrics of small-area mortality include the standardized mortality ratio (SMR), comparative mortality figure (CMF), and life expectancy (LE) [1, 2]. SMR, an indirect age-adjustment method, is generally known to have the advantages of relatively low variance, convenience in calculating, and the ability to be estimated even if the number of deaths or population is small or if the age-specific rate is not available [1, 3]. To be valid, a comparison of SMRs between groups should satisfy certain assumptions; specifically, the age distributions or the age-specific rate ratios of the groups to be compared should be similar [4–11]. However, controversy remains regarding to whether these assumptions must be strictly satisfied and whether these assumptions are of practical value [3, 12–14]. CMF, a direct age-adjustment method, has the advantage of yielding comparisons that are more straightforward logically than SMR because its denominator uses the number of deaths in a standard population for comparisons [5, 15]. However, CMF generally has a higher variance than SMR and can be calculated only when the age-specific mortality rate is available [5, 13, 14]. LE is defined as the average lifespan of a newborn baby when current age-specific mortality rates are applied to the future. LE has the advantage of not requiring a standard population for its calculation, and it is an intuitively well-accepted measurement for researchers, as well as for the public and policy-makers [1, 6, 16].

A valid measurement of health levels by area would be a first step not only for exploring social determinants of health and proportionate allocation of health resources but also for public awareness and political debate about health inequalities [17]. In particular, studies have been conducted in small areas that can more precisely measure local health levels [18–20]. The range of the LE at the small-area level in New Zealand was 28.5 years, which was more extensive than 5.0 years at the regional level [21]. Previous Korean studies, which estimated nationwide mortality at the small-area level in Korea, generally analyzed national administrative data (NAD) and death certificate data and used SMR as the mortality metric [22, 23]. Those studies used SMR as mortality metrics because Statistics Korea provides information on administrative provinces and districts in the microdata of the Korean death certificates, but does not release information on a small area (*dong/eup/myeon*) considering the disclosure risk of personal information. Meanwhile, Statistics Korea publicly releases aggregate numbers of deaths for each small area via its website. However, the validity of using SMR, which estimates nationwide mortality, at the small-area level has not been empirically examined in previous Korean studies. It is necessary to compare age-specific rates between groups or group-specific mortality levels adjusted by a direct method to determine the validity of using SMR to compare mortality levels between populations [9].

The smallest administrative units in Korea consist of *dong, eup*, and *myeon*. The *dong* is the lowest-level administrative unit in mainly metropolitan areas. There is no clear population threshold for the dong. However, dong can be installed only in a metropolitan city with a population of over 1 million or city with a population over 50,000. It is, therefore, the most populous and most urbanized of the three categories. The *myeon* is the most subdivided administrative unit of rural areas, and *eup* is defined as an area more than 20,000 population, mostly urbanized*.* Urbanized areas of an urban-rural complex city or a central district in rural areas may also be designated as *eup*, even if the population and urbanization standard are not met. If the *myeon* area satisfies population and urbanization standards, it can be promoted to eup [24]. From hereafter, the *dong* area is referred to as metropolitan, the *eup* as urban, and the *myeon* as rural. Supplementary Table 1 shows the age distribution across metropolitan, urban, and rural areas in 2015. Metropolitan and urban areas had a similar population structure, with a relatively large proportion of inhabitants aged 15–64 years. Rural areas had a higher proportion of the elderly population than the other two types of areas, and the mean and median values of age were higher. As age structure varies by administrative unit type, mortality metrics may be vulnerable to bias in comparisons of small-area mortality levels across the country using SMR as the mortality metric.

In this study, we aimed to compare SMR, CMF, and LE as mortality metrics for small areas in Korea. First, we assumed that all 3371 small areas had the same age-specific mortality and compared the distribution of estimated SMRs. Second, we compared the rankings of mortality calculated using SMR, CMF, and LE. Lastly, we examined the ratio of CMF to SMR stratified by urbanity.

## Methods

### Data

In this study, we utilized the National Health Information Database (NHID) from 2013 to 2017. The NHID covers the entire population of Korea and is managed and provided by the National Health Insurance Service, Korea’s single health insurance provider. The NHID is composed of several databases [20]. The eligibility database, one of the databases in the NHID, contains sociodemographic information on the entire population of Korea, including parameters such as sex, age, residence, and income-based insurance premiums [25]. Death information is also collected individually in conjunction with death certificate data from Statistics Korea [25]. In a previous study, the numbers of population and deaths at the district level (the administrative level in Korea above the *dong/eup/myeon* level) in the national statistics database and the NHID were highly correlated [26]. Prior research compared the NHID with the NAD of the Ministry of Interior and Safety (MOIS) for calculating small-area level mortality [27]. The numbers of population and deaths were nearly identical between the two databases, and the estimated SMRs were correlated to a great extent in both sexes [22]. Thus, using the NHID to estimate small-area mortality is considered to be valid. One of the substantive strengths of using the NHID to calculate small-area mortality is the availability of age-specific mortality data in each small area [25], unlike what was possible when using the NAD and death certificate data in previous studies [17, 18]. This strength allowed us to measure small-area mortality metrics, not only with SMR, but also with CMF and LE.

As of 1 January of each year, we obtained the annual population in small areas in 5-year age groups (0, 1–4, 5–9, 10–14, …, 85+) from the NHID as aggregated data. The subjects were followed up for 1 year, and those who died by the end of the year were classified as deceased. If the subjects were foreigners or did not have any gender, age, or residence information, they were excluded from the analysis (1.4% of total NHID subjects), and most of those (99.8%) were foreigners.

### Unit of analysis

The unit of analysis in this study was the *dong/eup/myeon*, which typically had between 3850 and 21,886 inhabitants and 46 and 109 deaths as of 2017. The distribution of the numbers of population and deaths among all 3377 small areas in this study is presented in Supplementary Table 2. The median population for each small area of the NHID for 2013–2017 was 111,077, (IQR = 181,207), the minimum was 10,244, and the maximum was 1,476,696. The metropolitan area had a higher median population than the urban area, but the median number of deaths were smaller. The rural area had a smaller population and death numbers than the other two areas, especially the population. Previous studies have also used the *dong/eup/myeon* as the unit of analysis to calculate small-area mortality in Korea [17, 18]. Due to changes in administrative districts over time, we adjusted the unit of analysis by analyzing merged or split small areas as one unit for the entire study period. Since it is known that more than 5000 subjects are required to calculate a stable LE [6], areas with an average population of less than 1000 per year were merged with adjacent areas. Finally, this study reclassified the 3500 *dong, eup*, and *myeon* areas as of 31 December 2017 to 3377 [28]. As of 31 December 2017, 8 out of 3500 small areas were excluded from the analysis as they are civilian access control areas for military purposes. There were 26 small areas with an average population of less than 1000 during the study period, and all other adjustments have been made due to administrative changes. The more detailed description of adjusting the unit of analysis can be found in another study [27]. Deidentified numbers were assigned to avoid stigma for small areas found to have high mortality rates [29].

### Statistical analysis

We estimated the SMR, CMF, and LE in all small areas in Korea. In this study, only age was considered to be a confounder of the association between areas and mortality, and was adjusted in the calculation of mortality metrics. Data from 2013 to 2017 consisted of a total of 64,163 (3377 small areas × 19 age bands) cells. Of those, 15,296 (23.8%) cells had 0 counts for deaths. A total of 6871 (17.8%) out of 38,114 cells had 0 counts for deaths in the metropolitan areas (*n* = 2006). In 221 urban areas, 691 (16.5%) out of 4199 cells had 0 counts for deaths, while rural area (*n* = 1150) had 0 counts for deaths in 7734 (35.4%) of 21,850 cells.

We used Eq. (1) to calculate SMR by dividing the number of observed deaths in a small area by the expected number of deaths. The expected deaths were estimated by multiplying the age-specific population in the small area by the age-specific mortality rate of the standard population. The standard population was the total population of this study.

1 $$ {\mathrm{SMR}}_r=\frac{\sum number\kern0.17em of\kern0.17em observed\kern0.17em deaths\kern0.17em in\kern0.17em each\kern0.17em small\kern0.17em area}{\sum \exp ected\kern0.17em number\kern0.17em of\kern0.17em deaths\kern0.17em in\kern0.17em each\kern0.17em small\kern0.17em area}=\frac{\sum_i{d}_{ir}}{\sum_i{t}_{ir}\left(\frac{D_i}{T_i}\right)} $$

Where *T*_*i*_ = age-specific population of standard population, *D*_*i*_ = age-specific number of deaths of standard population, *t*_*ir*_ = age-specific population of each small area, *d*_*ir*_ = age-specific number of deaths of each small area. *r* = small area, *i* = 5-year age group.

We followed the method presented in the previous study for calculating the standard error (SE) and 95% confidence interval (CI) of SMR [5].

2 $$ \mathrm{SE}\left(\mathrm{logSMR}\right)=\frac{\mathrm{SE}\left(\mathrm{SMR}\right)}{\mathrm{SMR}}=\frac{1}{\sqrt{d_r}} $$

3 $$ 95\%\mathrm{CI}:\kern0.5em \frac{\mathrm{SMR}}{\exp \left(\frac{1.96}{\sqrt{d_r}}\right)}\ \mathrm{to}\ \mathrm{SMR}\times \exp \left(\frac{1.96}{\sqrt{d_r}}\right) $$

CMF was calculated by dividing the expected number of deaths in the standard population by the number of observed deaths in the standard population. The expected number of deaths in the standard population was calculated by multiplying the age-specific mortality of each small area by the age-specific population in the standard population. The standard population used in the calculation of CMF was also the total population of this study. The Eq. (4) was used to calculate CMF.

4 $$ {\mathrm{CMF}}_{\mathrm{r}}=\frac{\sum \mathrm{expected}\kern0.17em \mathrm{number}\kern0.17em \mathrm{of}\kern0.17em \mathrm{deaths}\kern0.17em \mathrm{in}\kern0.17em \mathrm{standard}\kern0.17em \mathrm{population}}{\sum \mathrm{number}\kern0.17em \mathrm{of}\kern0.17em \mathrm{observed}\kern0.17em \mathrm{deaths}\kern0.17em \mathrm{in}\kern0.17em \mathrm{standard}\kern0.17em \mathrm{population}}=\frac{\sum_i{T}_i\left(\frac{d_{ir}}{t_{ir}}\right)}{\sum_i{D}_i} $$

We used Eqs. (5), (6), and (7) to estimate the SE and 95% CI of CMF [5].

5 $$ \mathrm{SE}\left(\mathrm{CMF}\right)=\frac{\sqrt{\sum_i{T}_i^2\frac{d_i}{n_i^2}}}{\sum_i{D}_i} $$

6 $$ \mathrm{SE}\left(\mathrm{logCMF}\right)=\frac{\mathrm{SE}\left(\mathrm{CMF}\right)}{\mathrm{CMF}} $$

7 $$ 95\%\mathrm{CI}:\kern0.5em \frac{\mathrm{CMF}}{\exp \left[\frac{1.96\times \mathrm{SE}\left(\mathrm{CMF}\right)}{\mathrm{CMF}}\right]}\ \mathrm{to}\ \mathrm{CMF}\times \exp \left[\frac{1.96\times \mathrm{SE}\left(\mathrm{CMF}\right)}{\mathrm{CMF}}\right] $$

We multiplied the calculated values of SMR and CMF by 100 to help readers understand more intuitively and to provide more detailed information [5, 30].

LE is often calculated by a deterministic approach [31]. Sampling variation is not an essential issue when calculating LE at national or regional levels [1]. However, when calculating LE at a small-area level, it is necessary to consider sampling variation according to the occurrence of stochastic variation over time [1, 16]. The calculation of the SE of LE can also answer the question of how many years of data must be combined to achieve the appropriate level of precision [1]. Chiang presumed that death numbers were distributed binomially, calculated the SE of the probability of dying in the interval, and linked it to the LE calculation in a previous study (as cited in [32]). Eayres and Williams contended that both assumptions—that deaths have a binomial distribution and a Poisson distribution—showed a high level of agreement in the results, but in the analysis of LE at the small-area level, they insisted that it would be preferable to assume a binomial distribution [33]. We performed Monte Carlo simulations using the probability of dying from an abridged life table to generate a binomial distribution of death numbers [1, 32]. The simulation was performed 10,000 times for each small area. We used it for the LE calculation and generated the LE distribution. The mean value of the distribution for a small area was defined as its LE. The 2.5th and 97.5th percentiles of the distribution were defined as the lower and upper limits of the CI of LE, respectively. No imputation was conducted even if the number of deaths for a specific age band was 0 [33, 34]. There was no small area where the number of deaths in the final age band (85+) was 0.

We set up a hypothetical situation with the same age-specific mortality rates across all small areas, applying the national age-specific mortality rates in 2015 to calculate SMR and to compare its distributions by urbanity. We also compared the ranking of areas by SMR, CMF, and LE, from the highest to lowest and from the lowest to highest. Lastly, we examined the ratio of CMF to SMR stratified by urbanity.

## Results

Table 1 presents the number of population and deaths, the age-specific mortality of the study subjects according to 5-year age groups, and the age-specific mortality ratios (MRs) in small areas. The total population was 254,194,174, and the number of deaths was 1,365,972. The vast majority of the total population (81.1%) resided in metropolitan areas, while the urban areas only accounted for 9.2%. The overall age-specific mortality was 537.4 per 100,000 population. The age group with the most substantial proportion of the population was 40–44 years, and the age group with the smallest proportion was 0 years. The age-specific mortality rate was highest among the ages of 85+, and lowest among the ages of 5–9. The age-specific mortality rate generally increased with age, except for the 0- to 4-year-old group. These patterns were true for metropolitan, urban, and rural areas. The age-specific patterns in age-specific mortality rates by urbanity also showed urban advantages. Supplementary Figure 1 graphically depicts the relationship between age and population proportion in MRs stratified by urbanity. Metropolitan and urban areas had relatively similar population age structures, while rural areas had a higher proportion of the elderly population than metropolitan and urban areas. The lowest mortality rate ratio was found in metropolitan areas, followed in order by urban and rural areas, but the magnitude of the difference decreased as age increased.

**Table 1:** Total and age-specific number of population and deaths, crude mortality, and mortality ratio by administrative type of small areas: data from the National Health Information Database of Korea, 2013–2017

	Total (*N* = 3377)			Metropolitan (dong) (*N* = 2006)				*Urban* (eup) (*N* = 221)				*Rural* (myeon) (*N* = 1150)			
Age group	Population	No. of deaths	Age-specific mortality (1)	Population	No. of deaths	Age-specific mortality (2)	MR (2)/(1)	Population	No. of deaths	Age-specific mortality (3)	MR (3)/(1)	Population	No. of deaths	Age-specific mortality (4)	MR (4)/(1)
Total	254,194,174 (100.0)	1,365,972 (100.0)	537.4	206,272,117 (100.0)	931,409 (100.0)	451.5	0.84	23,390,878 (100.0)	150,527 (100.0)	643.5	1.20	24,531,179 (100.0)	284,036 (100.0)	1157.9	2.15
0	2,214,887 (0.9)	1272 (0.1)	57.4	1,833,256 (0.9)	1008 (0.1)	55.0	0.96	229,968 (1.0)	162 (0.1)	70.4	1.23	151,663 (0.6)	102 (0.0)	67.3	1.17
1–4	9,274,613 (3.6)	1303 (0.1)	14.0	7,642,431 (3.7)	1041 (0.1)	13.6	0.97	995,867 (4.3)	153 (0.1)	15.4	1.10	636,315 (2.6)	109 (0.0)	17.1	1.22
5–9	11,652,184 (4.6)	1062 (0.1)	9.1	9,644,986 (4.7)	822 (0.1)	8.5	0.93	1,233,244 (5.3)	141 (0.1)	11.4	1.26	773,954 (3.2)	99 (0.0)	12.8	1.41
10–14	13,204,711 (5.2)	1243 (0.1)	9.4	11,056,969 (5.4)	977 (0.1)	8.8	0.94	1,297,202 (5.5)	146 (0.1)	11.3	1.20	850,540 (3.5)	120 (0.0)	14.1	1.50
15–19	16,563,705 (6.5)	4152 (0.3)	25.1	13,906,790 (6.7)	3321 (0.4)	23.9	0.95	1,498,029 (6.4)	432 (0.3)	28.8	1.15	1,158,886 (4.7)	399 (0.1)	34.4	1.37
20–24	17,232,200 (6.8)	5858 (0.4)	34.0	14,555,771 (7.1)	4707 (0.5)	32.3	0.95	1,393,583 (6.0)	574 (0.4)	41.2	1.21	1,282,846 (5.2)	577 (0.2)	45.0	1.32
25–29	15,914,203 (6.3)	7369 (0.5)	46.3	13,547,788 (6.6)	5925 (0.6)	43.7	0.94	1,229,154 (5.3)	645 (0.4)	52.5	1.13	1,137,261 (4.6)	799 (0.3)	70.3	1.52
30–34	19,170,329 (7.5)	11,929 (0.9)	62.2	16,248,390 (7.9)	9600 (1.0)	59.1	0.95	1,666,013 (7.1)	1178 (0.8)	70.7	1.14	1,255,926 (5.1)	1151 (0.4)	91.6	1.47
35–39	19,696,165 (7.7)	16,739 (1.2)	85.0	16,527,896 (8.0)	13,286 (1.4)	80.4	0.95	1,857,106 (7.9)	1735 (1.2)	93.4	1.10	1,311,163 (5.3)	1718 (0.6)	131.0	1.54
40–44	22,106,883 (8.7)	28,489 (2.1)	128.9	18,515,790 (9.0)	22,280 (2.4)	120.3	0.93	2,038,374 (8.7)	3041 (2.0)	149.2	1.16	1,552,719 (6.3)	3168 (1.1)	204.0	1.58
45–49	21,658,451 (8.5)	43,425 (3.2)	200.5	17,962,659 (8.7)	33,253 (3.6)	185.1	0.92	1,922,720 (8.2)	4667 (3.1)	242.7	1.21	1,773,072 (7.2)	5505 (1.9)	310.5	1.55
50–54	21,326,054 (8.4)	64,938 (4.8)	304.5	17,325,139 (8.4)	49,243 (5.3)	284.2	0.93	1,868,523 (8.0)	6708 (4.5)	359.0	1.18	2,132,392 (8.7)	8987 (3.2)	421.5	1.38
55–59	18,851,663 (7.4)	80,760 (5.9)	428.4	14,955,720 (7.3)	60,469 (6.5)	404.3	0.94	1,658,447 (7.1)	8205 (5.5)	494.7	1.15	2,237,496 (9.1)	12,086 (4.3)	540.2	1.26
60–64	13,372,783 (5.3)	84,431 (6.2)	631.4	10,306,291 (5.0)	62,014 (6.7)	601.7	0.95	1,211,282 (5.2)	8826 (5.9)	728.6	1.15	1,855,210 (7.6)	13,591 (4.8)	732.6	1.16
65–69	10,247,936 (4.0)	101,391 (7.4)	989.4	7,661,548 (3.7)	72,880 (7.8)	951.2	0.96	970,395 (4.1)	10,635 (7.1)	1095.9	1.11	1,615,993 (6.6)	17,876 (6.3)	1106.2	1.12
70–74	8,841,402 (3.5)	158,147 (11.6)	1788.7	6,217,726 (3.0)	107,739 (11.6)	1,732.8	0.97	902,932 (3.9)	17,550 (11.7)	1943.7	1.09	1,720,744 (7.0)	32,858 (11.6)	1909.5	1.07
75–79	6,576,957 (2.6)	214,395 (15.7)	3259.8	4,334,421 (2.1)	138,473 (14.9)	3,194.7	0.98	717,560 (3.1)	24,647 (16.4)	3434.8	1.05	1,524,976 (6.2)	51,275 (18.1)	3362.3	1.03
80–84	3,824,820 (1.5)	225,422 (16.5)	5893.7	2,443,707 (1.2)	142,961 (15.3)	5,850.2	0.99	427,183 (1.8)	25,962 (17.2)	6077.5	1.03	953,930 (3.9)	56,499 (19.9)	5922.8	1.00
85+	2,464,228 (1.0)	31,3647 (23.0)	12,728.0	1,584,839 (0.8)	201,410 (21.6)	12,708.5	1.00	273,296 (1.2)	35,120 (23.3)	12,850.5	1.01	606,093 (2.5)	77,117 (27.2)	12,723.6	1.00

*Notes*. *MR* mortality ratio. Crude mortality was defined as the number of deaths per 100,000 population

**Fig. 1:**
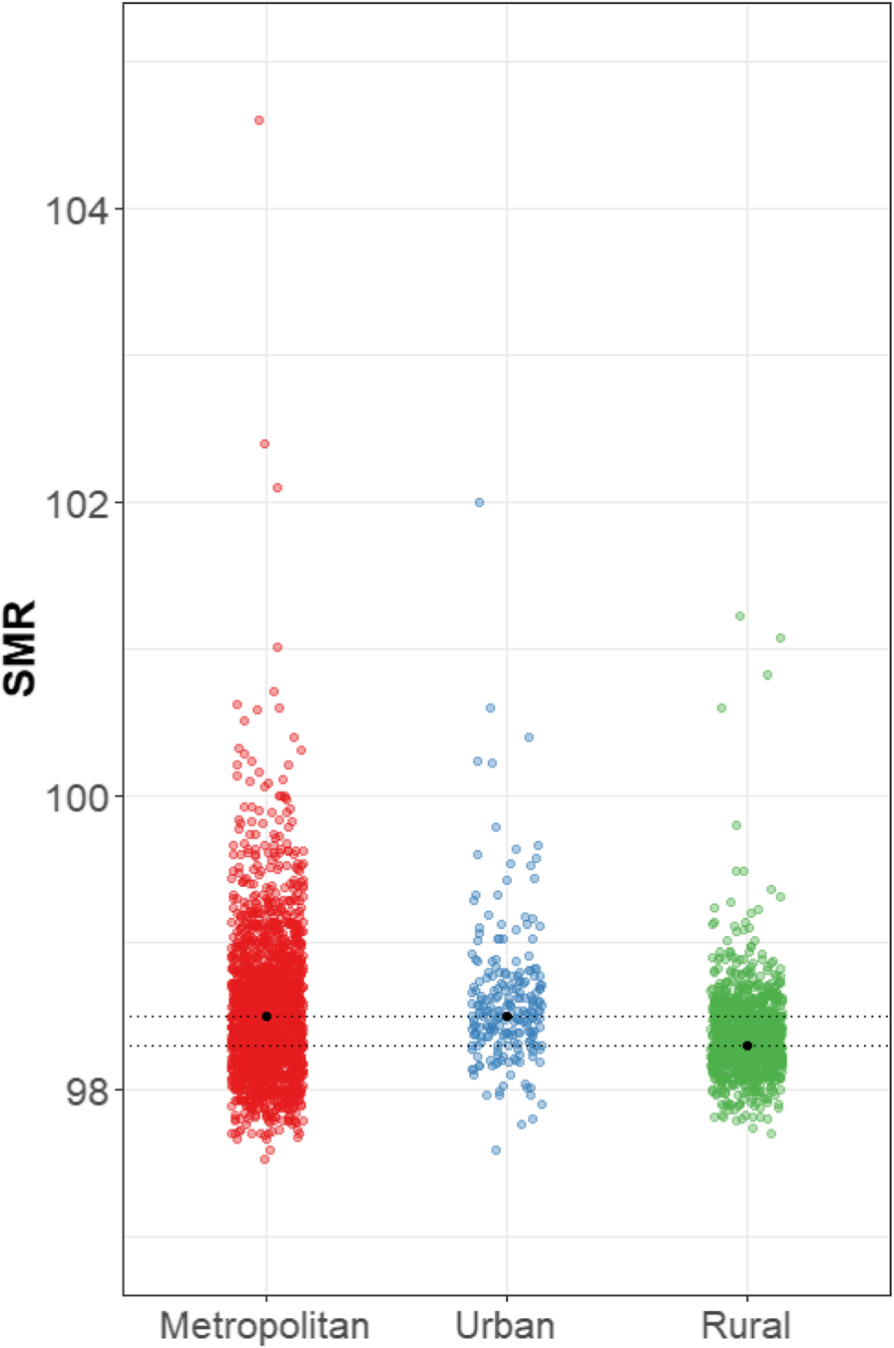
Comparison of SMRs according to the urbanity of 3371 small-areas under the hypothetical condition of equal age-specific mortality across the country: findings from the National Health Information Database of Korea, 2013–2017. *Notes*. SMR = standardized mortality ratio. SMR and CMF were rescaled by multiplying by 100. Metropolitan corresponds to *dong*, urban to *eup*, and rural to *myeon*

Figure 1 and Supplementary Table 3 show the hypothetical distribution of SMRs by urbanity, assuming that all small areas have the same age-specific mortality rates. Ideally, all small areas should have an equal value of SMR, but variation in SMR was observed. The median SMR in metropolitan, urban, and rural areas was 98.5, 98.5, and 98.3, respectively. The interquartile range (IQR) was 0.6 in metropolitan areas, which exceeded that observed in the other two types of areas. The maximum SMR was also the highest in metropolitan areas, with 104.6 among metropolitan areas, 102.0 among urban areas, and 101.2 among rural areas. In general, more small areas with a high SMR were distributed among the metropolitan areas. The minimum value was not meaningfully different across the three types of areas.

Table 2 shows the ranking of SMR in descending order across all small areas in comparison with the ranking of CMF and LE. When SMR was ranked in descending order, 22 of the top 30 small areas were metropolitan areas. The difference between SMR and LE was greater than the difference between CMF and LE. Supplementary Table 4 shows the ordered ranking of CMF in descending order in comparisons with the ranking of SMR and LE. In the ranking using CMF, only 7 of the 30 small areas were metropolitan areas. Table 3 shows the ranking of SMR in ascending order in comparison with the ranking of CMF. All 30 of the top 30 small areas were metropolitan areas, and smaller differences with CMF or LE were found in comparison to the top 30 small areas with high mortality. Supplementary Table 5 shows the top 30 small areas ranked by CMF in ascending order in comparison with the rankings of SMR and LE. Similarly, all 30 of these small areas were metropolitan areas, and the ranking of CMF did not differ notably according to the ranking of SMR or LE.

**Table 2:** Comparison of the top 30 small areas with high mortality as measured by SMR with their results for CMF and LE: findings from the National Health Information Database of Korea, 2013–2017

ID	Type	SMR (95% CI)	CMF (95% CI)	LE (95% CI)	SMR rank (highest to lowest) (1)	CMF rank (highest to lowest) (2)	LE rank (lowest to highest) (3)	(1)–(2)	(1)–(3)
862	*Rural*	181.8 (166.1,198.9)	189.0 (172.6,207.0)	74.8 (73.6,76.1)	1	1	3	0	− 2
778	*Metropolitan*	157.6 (139.4,178.2)	164.3 (144.6,186.7)	76.1 (74.1,78.1)	2	3	7	− 1	− 5
2952	*Metropolitan*	153.9 (143.7,164.8)	156.5 (145.8,168.0)	77.2 (76.1,78.3)	3	6	32	− 3	− 29
112	*Rural*	153.8 (141.7,166.9)	164.3 (151.1,178.8)	76.5 (75.3,77.6)	4	2	13	2	− 9
2302	*Metropolitan*	151.0 (137.7,165.6)	148.4 (134.9,163.3)	78.4 (77.3,79.3)	5	17	78	− 12	− 73
1248	*Metropolitan*	148.3 (133.3,165.0)	142.4 (127.8,158.6)	79.0 (77.6,80.4)	6	39	145	− 33	− 139
2934	*Metropolitan*	144.7 (134.2,156.0)	145.5 (134.7,157.2)	78.4 (77.4,79.3)	7	24	76	− 17	− 69
3266	*Rural*	143.8 (117.7,175.6)	153.1 (124.0,189.0)	76.4 (71.9,80.0)	8	13	10	− 5	− 2
1619	*Metropolitan*	141.3 (128.9,154.9)	133.5 (120.9,147.3)	79.1 (78.1,80.2)	9	134	167	− 125	− 158
3179	*Metropolitan*	140.1 (127.9,153.5)	124.3 (112.2,137.7)	79.4 (78.3,80.5)	10	379	238	− 369	− 228
2103	*Metropolitan*	140.0 (128.8,152.2)	137.9 (126.5,150.4)	79.2 (78.3,80.1)	11	77	185	− 66	− 174
3213	*Metropolitan*	139.5 (125.7,154.9)	139.1 (125.2,154.6)	78.1 (76.7,79.5)	12	65	57	− 53	− 45
1710	*Metropolitan*	139.2 (124.4,155.8)	137.6 (122.4,154.7)	79.0 (77.6,80.3)	13	82	141	− 69	− 128
1327	*Metropolitan*	139.1 (128.2,150.9)	139.1 (127.9,151.2)	78.7 (77.7,79.7)	14	66	104	− 52	− 90
1273	*Metropolitan*	137.9 (127.3,149.3)	139.3 (128.3,151.2)	79.0 (78.1,80.0)	15	63	152	− 48	− 137
1813	*Rural*	137.8 (115.3,164.7)	147.1 (120.5,179.4)	77.7 (74.0,81.1)	16	18	41	− 2	− 25
1228	*Metropolitan*	136.8 (127.9,146.3)	138.3 (129.3,148.0)	79.0 (78.3,79.8)	17	71	153	− 54	− 136
197	*Metropolitan*	135.9 (122.0,151.4)	144.3 (128.8,161.7)	78.1 (76.6,79.5)	18	29	56	− 11	− 38
1993	*Rural*	135.5 (119.4,153.8)	136.4 (118.7,156.9)	78.6 (75.6,80.9)	19	92	92	− 73	− 73
2754	*Metropolitan*	135.4 (122.6,149.5)	140.6 (127.0,155.6)	79.2 (78.1,80.3)	20	53	181	− 33	− 161
3263	*Metropolitan*	135.4 (123.0,149.1)	140.4 (127.0,155.4)	79.6 (78.6,80.6)	21	55	279	− 34	− 258
1009	*Metropolitan*	135.4 (122.4,149.8)	136.8 (123.1,151.9)	78.4 (76.8,79.8)	22	89	73	− 67	− 51
961	*Metropolitan*	135.4 (125.4,146.2)	134.7 (124.7,145.5)	79.0 (77.8,80.2)	23	109	144	− 86	− 121
766	*Metropolitan*	135.4 (123.6,148.3)	131.9 (120.1,144.8)	79.3 (78.4,80.1)	24	174	201	− 150	− 177
2323	*Rural*	135.3 (117.6,155.6)	132.5 (114.9,152.8)	79.7 (78.1,81.3)	25	158	316	− 133	− 291
1421	*Metropolitan*	134.8 (124.4,146.1)	137.0 (126.1,148.9)	79.1 (78.2,80.0)	26	86	159	− 60	− 133
1244	*Rural*	134.6 (122.4,148.0)	141.8 (128.5,156.5)	78.3 (77.0,79.6)	27	42	72	− 15	− 45
1274	*Metropolitan*	134.6 (122.8,147.6)	134.8 (122.8,147.8)	78.8 (77.7,80.0)	28	108	117	− 80	− 89
1650	*Metropolitan*	134.4 (120.1,150.4)	139.5 (124.0,157.1)	79.1 (77.5,80.6)	29	62	161	− 33	− 132
488	*Rural*	134.3 (116.8,154.5)	154.5 (128.0,186.5)	79.1 (77.9,80.1)	30	10	157	20	− 127

*Notes*. *CI* confidence interval, *CMF* comparative mortality figure, *LE* life expectancy, *SMR* standardized mortality ratio

SMR and CMF were rescaled by multiplying by 100

Metropolitan corresponds to *dong*, urban to *eup*, and rural to *myeon*

**Table 3:** Comparison of top 30 small areas with low mortality as measured by SMR with their results for CMF and LE: findings from the National Health Information Database of Korea, 2013–2017

ID	Type	SMR (95% CI)	CMF (95% CI)	LE (95% CI)	SMR rank (lowest to highest) (1)	CMF rank (lowest to highest) (2)	LE rank (highest to lowest) (3)	(1)–(2)	(1)–(3)
1016	*Metropolitan*	51.4 (44.1,59.9)	49.4 (42.4,57.7)	89.8 (88.9,90.8)	1	1	2	0	− 1
231	*Metropolitan*	55.3 (48.2,63.5)	60.2 (52.1,69.4)	87.6 (86.5,88.7)	2	6	14	− 4	− 12
1270	*Metropolitan*	57.0 (50.2,64.7)	59.1 (51.9,67.4)	87.7 (86.8,88.6)	3	4	12	− 1	− 9
1864	*Metropolitan*	57.7 (52.5,63.4)	55.3 (50.3,60.8)	88.6 (88.0,89.3)	4	2	4	2	0
318	*Metropolitan*	57.8 (52.5,63.6)	58.2 (49.1,69.0)	87.7 (87.0,88.4)	5	3	3	2	2
1290	*Metropolitan*	57.8 (48.9,68.3)	60.7 (55.0,66.9)	89.2 (87.9,90.5)	6	10	11	− 4	− 5
3360	*Metropolitan*	58.5 (52.6,65.0)	60.5 (54.4,67.4)	87.5 (86.8,88.2)	7	8	17	− 1	− 10
3115	*Metropolitan*	59.8 (52.8,67.7)	60.7 (53.5,68.8)	87.6 (86.8,88.5)	8	9	13	− 1	− 5
1298	*Metropolitan*	60.0 (53.4,67.4)	61.0 (54.3,68.6)	87.8 (87.0,88.7)	9	11	8	− 2	1
2776	*Metropolitan*	60.3 (55.6,65.4)	59.5 (54.9,64.5)	87.9 (87.4,88.5)	10	5	6	5	4
502	*Metropolitan*	61.2 (54.5,68.7)	60.3 (53.6,67.8)	88.0 (87.0,89.0)	11	7	5	4	6
1273	*Metropolitan*	61.5 (55.1,68.7)	70.0 (62.2,78.8)	86.0 (85.2,86.7)	12	54	87	− 42	− 75
1871	*Metropolitan*	61.6 (56.3,67.5)	66.8 (60.8,73.3)	86.6 (86.0,87.3)	13	30	45	− 17	− 32
849	*Metropolitan*	62.4 (57.1,68.2)	66.8 (60.9,73.3)	86.7 (86.1,87.4)	14	31	40	− 17	− 26
3067	*Metropolitan*	62.5 (55.7,70.1)	63.8 (59.1,68.9)	87.4 (86.5,88.2)	15	17	21	− 2	− 6
1756	*Metropolitan*	62.5 (57.9,67.5)	65.3 (58.1,73.4)	87.0 (86.5,87.5)	16	23	31	− 7	− 15
311	*Metropolitan*	62.7 (57.4,68.5)	62.2 (56.9,68.0)	87.6 (86.9,88.2)	17	12	15	5	2
2665	*Metropolitan*	62.8 (55.3,71.4)	63.0 (55.4,71.6)	87.7 (86.7,88.6)	18	14	10	4	8
1371	*Metropolitan*	62.8 (57.7,68.4)	64.1 (58.8,69.9)	86.9 (86.3,87.4)	19	18	35	1	− 16
2232	*Metropolitan*	63.2 (57.2,69.9)	65.3 (59.0,72.4)	86.8 (86.1,87.5)	20	24	37	− 4	− 17
1480	*Metropolitan*	63.3 (58.2,68.9)	62.8 (56.1,70.2)	87.2 (86.6,87.9)	21	13	25	8	− 4
1111	*Metropolitan*	63.3 (56.6,70.8)	63.7 (58.3,69.5)	86.9 (86.2,87.7)	22	16	32	6	− 10
1269	*Metropolitan*	63.4 (56.7,70.9)	64.7 (57.5,72.8)	87.8 (86.8,88.7)	23	21	9	2	14
3214	*Metropolitan*	63.8 (57.0,71.4)	64.4 (57.5,72.3)	87.9 (87.0,88.7)	24	20	7	4	17
879	*Metropolitan*	64.1 (57.5,71.5)	66.2 (59.1,74.1)	86.7 (85.8,87.5)	25	26	43	− 1	− 18
1303	*Metropolitan*	64.2 (58.7,70.2)	63.1 (57.7,69.1)	87.4 (86.7,88.1)	26	15	19	11	7
2828	*Metropolitan*	64.4 (57.6,72.0)	64.3 (57.4,71.9)	87.2 (86.3,88.0)	27	19	26	8	1
2728	*Metropolitan*	64.8 (56.9,73.8)	64.8 (56.8,74.0)	87.6 (86.5,88.6)	28	22	16	6	12
1001	*Metropolitan*	65.1 (57.8,73.3)	69.3 (61.4,78.2)	86.3 (85.4,87.1)	29	51	61	− 22	− 32
2103	*Metropolitan*	65.2 (57.1,74.4)	66.2 (60.8,72.1)	86.7 (85.7,87.6)	30	27	27	3	3

*Notes. CI* confidence interval, *CMF* comparative mortality figure, *LE* life expectancy, *SMR* standardized mortality ratio

SMR and CMF were rescaled by multiplying by 100

Metropolitan corresponds to *dong*, urban to *eup*, and rural to *myeon*

**Fig. 2:**
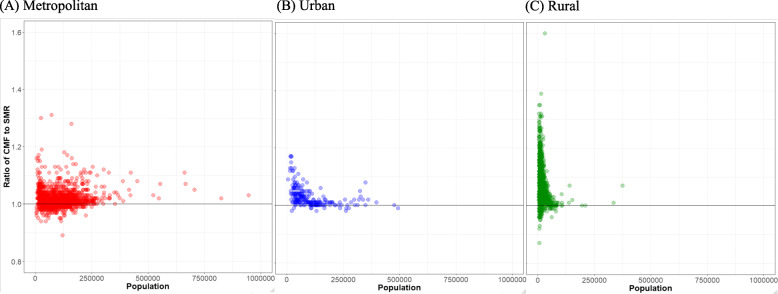
Scatter plots of the relationship between population and the ratio of small-area level CMF to SMR stratified by urbanity: findings from the National Health Information Database of Korea, 2013–2017. *Notes*. CMF = comparative mortality figure; SMR = standardized mortality ratio. SMR and CMF were rescaled by multiplying by 100. Metropolitan corresponds to *dong*, urban to *eup*, and rural to *myeon*

Figure 2 shows the ratio of CMF to SMR at the small-area level. In metropolitan and urban areas, the ratios of CMF to SMR were mostly less than 1.1, although there were differences according to population size. However, in rural areas, the ratio of CMF to SMR tended to be high, particularly in small areas with a small population. Supplementary Figure 2 presents the correlation coefficients [*r*] and scatter plots of SMR, CMF, and LE over all areas and stratified by urbanity. Overall, the magnitude of the correlation between CMF and LE was the largest (*r* = − 0.972), and the correlation between SMR and LE was the smallest (*r* = − 0.888). The correlations between SMR, CMF, and LE were strong in metropolitan areas, with the absolute magnitude of the correlation coefficients ranging from 0.967 to 0.984. In urban areas, the absolute magnitude of the correlation coefficients ranged from 0.884 to 0.973. In rural areas, the absolute magnitude of the correlation coefficients ranged from 0.733 to 0.932. In all stratified analyses, the less correlated indicators were SMR and LE.

## Discussion

This study compared mortality statistics measured by the SMR, CMF, and LE in all small areas of Korea, according to urbanity. When we used the hypothetical assumption that age-specific mortality would be equal in all small areas, metropolitan areas showed higher SMRs than rural areas. When we compared the ranking of areas by SMR with those of CMF and LE in ascending and descending order, we found notable differences in small areas with high mortality. However, no meaningful difference was found in the ranking of small areas with low mortality by SMR, CMF, or LE. The mismatch between SMR and CMF was driven by rural areas with small populations. The magnitudes of the correlations between CMF and LE were notably stronger than those between SMR and LE, and between SMR and CMF, especially in rural areas.

Two assumptions must be satisfied when comparing multiple SMRs [4–11]. First, homogeneous age-specific MRs across all strata are needed between the populations to be compared. This assumption requires that the MRs of the study populations to the standard population be homogeneous across all strata. The second assumption is that population structures need to be similar between the populations to be compared. However, it is not necessary to satisfy both assumptions simultaneously. Those assumptions are required because the SMR is a weighted average of age-specific MRs across strata groups [4]. There is a theoretical limitation to comparing SMRs since the expected number of deaths of each study population is used as the denominator, meaning that the denominators are different when comparing SMRs. The number of expected deaths in CMF is calculated by multiplying the age-specific mortality rate of each study population by the age-specific population of the standard population. Since the observed number of deaths of the standard population is used as the denominator in the calculation of CMF, CMF has at least a theoretical advantage and a better justification for use in comparisons [5]. As a consequence, CMF differs between populations being compared only when the age-specific MRs differ between the populations, whereas a difference in SMR can also be caused by differences in population structure [1].

Controversy has emerged regarding the bias induced by population age structure in calculating SMR in practice [1, 13, 35]. Gustafson argued that violation of these assumptions would not cause serious problems for practical purposes, although he presumed that the age structures would not be markedly different between populations [12]. Court and Cheng also contended that the results would be robust even if those assumptions were not satisfied [3]. However, some researchers have asserted that despite the many advantages of SMR, CMF should be used for comparisons [15], primarily due to the use of common denominators between populations to be compared [5]. Goldman and Brender concurred regarding the practical usefulness of SMR. However, they urged that CMF be used because in small area analyses, variations in population age structure could cause important biases in the results of SMR [14].

When the age-specific mortality rates of all small areas were assumed to be the national age-specific mortality rates in 2015, the formula for estimating SMR presented in the method section can be transformed as follows:

8 $$ \frac{\sum_i{t}_{ir}\left(\frac{D_i,2015}{T_i,2015}\right)}{\sum_i{t}_{ir}\left(\frac{D_i}{T_i}\right)} $$

Where *T*_*i*, 2015_ = national age-specific population in 2015, *D*_*i*, 2015_ = national age-specific number of deaths in 2015.

Even if the age-specific mortality rates were hypothesized as the same, the number of expected and observed deaths vary by small areas, depending on age structure. SMR decreases when the increase in the denominator (the sum of the expected number of deaths by age group) is greater than the increase in the numerator (the sum of observed deaths by age group) compared to the denominator and numerator in the areas with median SMR. When the decrease in the denominator is smaller than the decrease in the numerator, SMR also decreases. In reverse, SMR increases. However, when the changes in denominator and numerator cancel out, the SMR converges to the median value. Variance in SMR would be larger in areas with a high proportion of young age groups such as metropolitan areas due to small denominators. SMR also can be calculated by using the sum of the difference between the age-specific expected and observed deaths and the sum of the number of expected deaths as the follow equation.

9 $$ 1-\left[\frac{\sum_i{t}_{ir}\left(\frac{D_i}{T_i}\right)-{\sum}_i{t}_{ir}\left(\frac{D_{i,2015}}{T_{i,2015}}\right)}{\sum_i{t}_{ir}\left(\frac{D_i}{T_i}\right)}\right] $$

Supplementary Table 6 shows age structures, the number of expected and observed deaths in small areas where SMR shows minimum (study population 1), maximum (study population 2), and median (study population 3) values, respectively, when national age-specific mortality in 2015 is applied to all small areas. We also measured the contribution by age group to the sum of the differences between expected and observed deaths. In study population 1, older adults were more likely to contribute to the difference between observed and expected deaths, but in the 0, 1–4 age group where national age-specific mortality in 2015 was greater than age-specific mortality of the standard population (working toward increasing SMR), the contribution was relatively small compared to study population 3. This was because the proportion of the 0, 1–4 age group was smaller than that of the corresponding age group in the study population 3. Study population 2 had a smaller population and a relatively large proportion of younger people than study populations 1 and 3. The considerable contribution of the age group of 0 and 1–4 provided higher SMR than the median value. The small denominator would be sensitive to small differences in the numerator. Taken together, the SMR may be larger or smaller than the median value depending on the difference in the age-specific mortality between the study population and the standard population weighted by the age-specific population. In areas of low population, the variation was likely to be more significant because the sum of the expected number of deaths was usually smaller. The minimum values in metropolitan, urban, and rural areas were similar. These areas often had a higher proportion of older people, with higher expected death and smaller variance. However, the maximum and IQR values of SMR varied by the administrative type of small areas. In particular, larger variation and the highest maximum values were observed in metropolitan areas where the sum of the expected number of deaths was relatively small, with a large proportion of younger age groups. Furthermore, when using NHID from 2013 to 2017, 22 of the top 30 small areas were found to be metropolitan areas when SMR values were sorted in descending order. In contrast, when CMF values were sorted, only 7 of the top 30 small areas were metropolitan areas. Therefore, CMF or LE should be used rather than SMR to rank small area-specific mortality across the country or in regions with different population age structures. In any case, it is always necessary to present the CI with the results [35, 36].

SMR is a weighted average of the ratios of age-specific mortality of the population of interest to the age-specific mortality of the standard population, using the proportion of the population of interest by age as weight. Thus, SMR is affected by the MR, as well as the population age structure. Metropolitan and urban areas had a very similar population structure, and the differences in MRs were relatively small when the entire sample was set as the standard population. Furthermore, the magnitude of the MR was homogeneous across age groups in these two types of areas. The reason for these findings is thought to be that the standard population was set for the entire sample. Since the age standardization rate depends on the standard population, the selection of the standard population is vital [9]. A generally accepted standard population is one that does not differ significantly from the group to be compared in characteristics such as age and gender [7]. Szklo and Nieto provided several examples of standard populations, including a selection of the study group with the smallest population, the society that the study subjects jointly belong to, and the minimum-variance standard population [37]. In future studies, when measuring age-standardized mortality across the country, efforts should be made to select appropriate populations; for example, alternatives could include using the population of *eup* areas as the standard population or using a minimum-variance standard population. LE, which does not require a standard population to be selected, is also considered to be an appropriate method [1].

In rural areas, the ratio of CMF to SMR ranged from 0.87 to 1.60, which was a notably broader range than was observed for the other two types of areas (metropolitan 0.89–1.37, urban 0.98–1.17). In addition, the smaller the population, the farther the ratio was from 1. The correlations between SMR and CMF, and between SMR and LE were highest in metropolitan areas, followed in order by urban and rural areas. Since CMF was also most correlated with LE in metropolitan areas, followed in the same order by urban and rural areas, this may have been due to stochastic variation in age-specific mortality in areas with low populations when calculating CMF. However, the magnitudes of the correlation coefficients between CMF and LE were greater than those between SMR and LE. Furthermore, the range of the ratio of CMF to SMR in rural areas was wider than in the other two types of areas with similar populations. Thus, it is likely that these results stemmed from the failure to satisfy the assumptions needed to estimate valid SMRs.

This study has limitations. This study did not reflect spatial trends in the analysis. Previous studies showed that estimates could be biased if structural spatial effects are not taken into account [38]. In the same context, disease mapping studies often use the Bayesian random effect model to borrow information from adjacent areas [38, 39]. However, this study examined whether two assumptions for valid SMR calculations are met in a nationwide SMR calculation. Because small areas with similar age structures or MRs are distributed in adjacent regions according to urbanity, it may be meaningful to compare them in the frequentist view.

## Conclusions

This study conducted a comparison of small-area level SMR, CMF, and LE in Korea. When we hypothesized that the age-specific mortality rates of all small areas would be the same, the results showed that the median and minimum values of SMR did not differ notably according to urbanity. However, there were more small areas with a high SMR in metropolitan areas. The proportion of metropolitan areas among the top 30 areas with the highest SMRs was higher than the proportion obtained by calculating CMF. Furthermore, the ranking obtained using LE showed a larger difference relative to the ranking obtained using SMR than it did to the ranking using CMF. The relative difference between SMR and CMF was large in rural areas, especially in areas with a small population size. This most likely occurred because, in addition to stochastic variations in CMF calculation due to the small populations, the two necessary assumptions for SMR calculations were not fully met. These results indicate that using SMR to compare mortality in small areas across the country is likely to lead to bias, suggesting that it is more appropriate to compare using CMF or LE if age-specific mortality data are available. When comparing mortality using SMR, the study area should cover small areas where the population structure is not significantly different, and an appropriate standard population should be selected. A valid measurement of small-area health levels would enable exploring social determinants of health and proportionate health resource allocation as well as raising public awareness of health inequalities across the country and triggering political debate. As shown in this study, if bias is present in the measurement of mortality in small areas, particularly, with high mortality, it is likely to interfere with the allocation of health resources or policy decision-making. Furthermore, mismeasurement of small-area mortalities could lead to false public awareness or political debate. It is necessary to establish a database to utilize mortality and morbidity metrics that reflect the age structure and characteristics of the area for measuring small-area health levels validly.

## Supplementary information

**Additional file 1: Figure S1**. Age distribution and age-specific mortality ratios by urbanity with the standard population: findings from the National Health Information Database of Korea, 2013–2017. **Figure S2**. Scatter plots and correlation coefficients [r] for SMR, CMF, and LE over all areas and stratified by urbanity: findings from the National Health Information Database of Korea, 2013–2017. **Table S1**: Population size and age structure by urbanity: data from the 2015 Population Census of Korea. **Table S2**: Distribution of numbers of population and deaths among 3,377 small-areas in Korea: findings from the National Health Information Database of Korea, 2013–2017. **Table S3**: Distribution of SMRs according to urbanity when the age-specific mortality rate was hypothesized to be the same in all small-areas: findings from the National Health Information Database of Korea, 2013–2017. **Table S4**: Comparison of the top 30 small-areas with high mortality as measured by CMF with their results for SMR and LE: findings from the National Health Information Database of Korea, 2013–2017. **Table S5**: Comparison of the top 30 small-areas with low mortality as measured by CMF with their results for SMR and LE: findings from the National Health Information Database of Korea, 2013–2017. **Table S6**: Age structure, observed and expected deaths using national age-specific mortality in 2015 and age-specific mortality of the standard population, and their differences in small-areas with the minimum, maximum, and median SMR: findings from the National Health Information Database of Korea, 2013–2017

## Data Availability

The data that support the findings of this study are available from the National Health Insurance Service of Korea but restrictions apply to the availability of these data, which were used under license for the current study, and so are not publicly available. Data are however available from the authors upon reasonable request and with permission of the National Health Insurance Service.

## Abbreviations

CI: Confidence interval
CMF: Comparative mortality figure
IQR: Interquartile ratio
LE: Life expectancy
MR: Mortality ratio
NAD: National Administrative Data
NHID: National Health Information Database
SE: Standard error
SMR: Standardized mortality ratio
