# Supplementary material for: Comparison of three small-area mortality metrics according to urbanity in Korea: the standardized mortality ratio, comparative mortality figure, and life expectancy
Source: Popul Health Metr. 2020 Jul 3;18:3. doi: 10.1186/s12963-020-00210-7 (PMC7333282; doi:10.1186/s12963-020-00210-7)
Supplement: Supplementary file 1 — Additional file 1: Figure S1. Age distribution and age-specific mortality ratios by urbanity with the standard population: findings from the National Health Information Database of Korea, 2013–2017. Figure S2. Scatter plots and correlation coefficients [r] for SMR, CMF, and LE over all areas and stratified by urbanity: findings from the National Health Information Database of Korea, 2013–2017. Table S1: Population size and age structure by urbanity: data from the 2015 Population Census of Korea. Table S2: Distribution of numbers of population and deaths among 3,377 small-areas in Korea: findings from the National Health Information Database of Korea, 2013–2017. Table S3: Distribution of SMRs according to urbanity when the age-specific mortality rate was hypothesized to be the same in all small-areas: findings from the National Health Information Database of Korea, 2013–2017. Table S4: Comparison of the top 30 small-areas with high mortality as measured by CMF with their results for SMR and LE: findings from the National Health Information Database of Korea, 2013–2017. Table S5: Comparison of the top 30 small-areas with low mortality as measured by CMF with their results for SMR and LE: findings from the National Health Information Database of Korea, 2013–2017. Table S6: Age structure, observed and expected deaths using national age-specific mortality in 2015 and age-specific mortality of the standard population, and their differences in small-areas with the minimum, maximum, and median SMR: findings from the National Health Information Database of Korea, 2013–2017 [file 12963_2020_210_MOESM1_ESM.docx]

Supplementary Table 1. Population size and age structure by urbanity: data from the 2015 Population Census of Korea

|  | Metropolitan | Urban | Rural |
| --- | --- | --- | --- |
| Total population | 41,677,695 | 4,616,802 | 4,774,878 |
| Age group |  |  |  |
| Under 15 (%) | 14.0 | 15.3 | 9.0 |
| 15–64 (%) | 74.8 | 70.4 | 64.3 |
| 65+ (%) | 11.2 | 14.4 | 26.7 |
| Mean (yr) | 39.4 | 40.4 | 48.4 |
| Median (yr) | 40.1 | 41.1 | 51.2 |

*Notes.* Metropolitan corresponds to *dong*, urban to *eup*, and rural to *myeon*.

Supplementary Table 2. Distribution of numbers of population and deaths among 3,377 small-areas in Korea: findings from the National Health Information Database of Korea, 2013–2017.

|  | No. of population | | | No. of deaths | | |
| --- | --- | --- | --- | --- | --- | --- |
|  | Median  (IQR) | Minimum | Maximum | Median  (IQR) | Minimum | Maximum |
| Total | 111,077  (181,207) | 10,244 | 1,476,696 | 677  (547) | 50 | 4,956 |
| Metropolitan | 184,637  (157,904) | 11,991 | 1,476,696 | 827  (516) | 50 | 4,956 |
| Urban | 156,131  (172,083) | 12,165 | 915,262 | 1,212  (742) | 181 | 4,479 |
| Rural | 32,307  (25,027) | 10,244 | 460,205 | 443  (266) | 94 | 2,341 |

*Notes.* IQR = Interquartile range.

Metropolitan corresponds to *dong*, urban to *eup*, and rural to *myeon*.


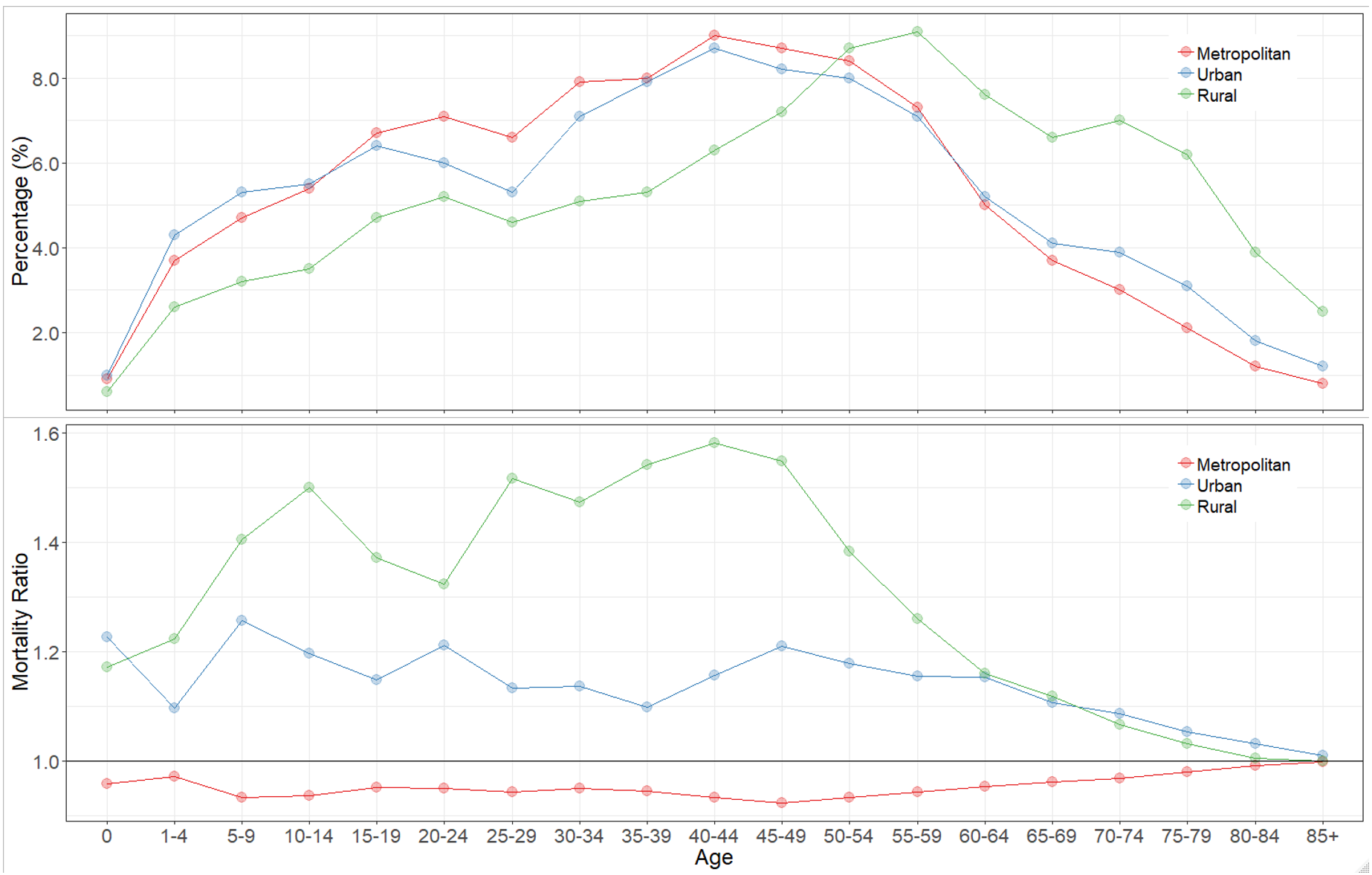


Supplementary Figure 1. Age distribution and age-specific mortality ratios by urbanity with the standard population: findings from the National Health Information Database of Korea, 2013–2017.

Supplementary Table 3. Distribution of SMRs according to urbanity when the age-specific mortality rate was hypothesized to be the same in all small-areas: findings from the National Health Information Database of Korea, 2013–2017.

|  | Median | IQR | Min | Max |
| --- | --- | --- | --- | --- |
| Metropolitan | 98.5 | 0.6 | 97.5 | 104.6 |
| Urban | 98.5 | 0.3 | 97.6 | 102.0 |
| Rural | 98.3 | 0.3 | 97.7 | 101.2 |

*Notes.* IQR = Interquartile range; Max = Maximum; Min = Minimum.

SMF was rescaled by multiplying by 100.

Metropolitan corresponds to *dong*, urban to *eup*, and rural to *myeon*.

Supplementary Table 4. Comparison of the top 30 small-areas with high mortality as measured by CMF with their results for SMR and LE: findings from the National Health Information Database of Korea, 2013–2017

| ID | Type | CMF (95% CI) | | SMR (95% CI) | | LE (95% CI) | CMF rank (highest to lowest)  (1) | SMR rank (highest to lowest) (2) | LE rank (lowest to highest) (3) | (1) - (2) | (1) - (3) |
| --- | --- | --- | --- | --- | --- | --- | --- | --- | --- | --- | --- |
| 862 | *Rural* | 189.0 (172.6,207.0) | 181.8 (166.1,198.9) | | 74.8 (73.6,76.1) | | 1 | 1 | 3 | 0 | -2 |
| 112 | *Rural* | 164.3 (151.1,178.8) | 153.8 (141.7,166.9) | | 76.1 (74.1,78.1) | | 2 | 4 | 13 | -2 | -11 |
| 778 | *Metropolitan* | 164.3 (144.6,186.7) | 157.6 (139.4,178.2) | | 77.2 (76.1,78.3) | | 3 | 2 | 7 | 1 | -4 |
| 1573 | *Rural* | 159.5 (133.9,189.9) | 121.8 (108.1,137.2) | | 76.5 (75.3,77.6) | | 4 | 227 | 26 | -223 | -22 |
| 2685 | *Rural* | 158.3 (126.2,198.7) | 127.9 (106.5,153.5) | | 78.4 (77.3,79.3) | | 5 | 102 | 4 | -97 | 1 |
| 2952 | *Metropolitan* | 156.5 (145.8,168.0) | 153.9 (143.7,164.8) | | 79.0 (77.6,80.4) | | 6 | 3 | 32 | 3 | -26 |
| 2326 | *Rural* | 156.5 (128.2,191.0) | 122.8 (106.4,141.7) | | 78.4 (77.4,79.3) | | 7 | 194 | 28 | -187 | -21 |
| 1757 | *Rural* | 156.2 (137.5,177.4) | 133.6 (119.3,149.6) | | 76.4 (71.9,80.0) | | 8 | 36 | 19 | -28 | -11 |
| 260 | *Rural* | 155.1 (128.5,187.2) | 133.1 (114.6,154.6) | | 79.1 (78.1,80.2) | | 9 | 40 | 25 | -31 | -16 |
| 1593 | *Rural* | 154.5 (128.0,186.5) | 134.3 (116.8,154.5) | | 79.4 (78.3,80.5) | | 10 | 30 | 5 | -20 | 5 |
| 562 | *Metropolitan* | 154.1 (133.0,178.6) | 131.6 (115.0,150.6) | | 79.2 (78.3,80.1) | | 11 | 54 | 27 | -43 | -16 |
| 2221 | *Rural* | 153.5 (125.9,187.3) | 121.2 (104.9,140.1) | | 78.1 (76.7,79.5) | | 12 | 249 | 8 | -237 | 4 |
| 3266 | *Rural* | 153.1 (124.0,189.0) | 143.8 (117.7,175.6) | | 79.0 (77.6,80.3) | | 13 | 8 | 10 | 5 | 3 |
| 1739 | *Metropolitan* | 152.1 (130.3,177.6) | 132.8 (117.5,150.1) | | 78.7 (77.7,79.7) | | 14 | 43 | 14 | -29 | 0 |
| 1033 | *Rural* | 149.5 (124.4,179.7) | 118.5 (103.8,135.3) | | 79.0 (78.1,80.0) | | 15 | 353 | 12 | -338 | 3 |
| 3143 | *Rural* | 149.3 (119.7,186.4) | 117.4 (99.7,138.2) | | 77.7 (74.0,81.1) | | 16 | 401 | 23 | -385 | -7 |
| 2302 | *Metropolitan* | 148.4 (134.9,163.3) | 151.0 (137.7,165.6) | | 79.0 (78.3,79.8) | | 17 | 5 | 78 | 12 | -61 |
| 1813 | *Rural* | 147.1 (120.5,179.4) | 137.8 (115.3,164.7) | | 78.1 (76.6,79.5) | | 18 | 16 | 41 | 2 | -23 |
| 2812 | *Rural* | 146.2 (122.5,174.4) | 124.8 (108.6,143.4) | | 78.6 (75.6,80.9) | | 19 | 149 | 31 | -130 | -12 |
| 1670 | *Rural* | 146.1 (126.6,168.7) | 130.4 (115.9,146.7) | | 79.2 (78.1,80.3) | | 20 | 67 | 36 | -47 | -16 |
| 680 | *Rural* | 146.0 (128.4,166.1) | 133.6 (119.7,149.1) | | 79.6 (78.6,80.6) | | 21 | 37 | 52 | -16 | -31 |
| 1398 | *Rural* | 145.9 (119.0,178.8) | 127.8 (110.3,148.0) | | 78.4 (76.8,79.8) | | 22 | 105 | 2 | -83 | 20 |
| 2820 | *Rural* | 145.6 (127.4,166.4) | 128.5 (114.2,144.6) | | 79.0 (77.8,80.2) | | 23 | 94 | 48 | -71 | -25 |
| 2934 | *Metropolitan* | 145.5 (134.7,157.2) | 144.7 (134.2,156.0) | | 79.3 (78.4,80.1) | | 24 | 7 | 76 | 17 | -52 |
| 2433 | *Rural* | 145.5 (120.7,175.3) | 119.1 (103.8,136.7) | | 79.7 (78.1,81.3) | | 25 | 330 | 15 | -305 | 10 |
| 2747 | *Rural* | 144.8 (127.5,164.5) | 127.2 (113.8,142.2) | | 79.1 (78.2,80.0) | | 26 | 114 | 22 | -88 | 4 |
| 966 | *Rural* | 144.7 (127.9,163.6) | 130.5 (116.8,145.8) | | 78.3 (77.0,79.6) | | 27 | 65 | 65 | -38 | -38 |
| 2638 | *Rural* | 144.5 (123.0,169.8) | 119.4 (105.6,135.1) | | 78.8 (77.7,80.0) | | 28 | 315 | 9 | -287 | 19 |
| 197 | *Metropolitan* | 144.3 (128.8,161.7) | 135.9 (122.0,151.4) | | 79.1 (77.5,80.6) | | 29 | 18 | 56 | 11 | -27 |
| 2651 | *Rural* | 144.3 (120.4,172.9) | 110.5 (97.3,125.5) | | 79.1 (77.9,80.1) | | 30 | 905 | 37 | -875 | -7 |

*Notes.* CI = Confidence interval; CMF = Comparative mortality figure; LE = Life expectancy; SMR = Standardized mortality ratio.

SMR and CMF were rescaled by multiplying by 100.

Metropolitan corresponds to *dong*, urban to *eup*, and rural to *myeon*.

Supplementary Table 5. Comparison of the top 30 small-areas with low mortality as measured by CMF with their results for SMR and LE: findings from the National Health Information Database of Korea, 2013–2017

| ID | Type | CMF (95% CI) | SMR (95% CI) | LE (95% CI) | SMR rank (lowest to highest) (1) | CMF rank  (lowest to highest) (2) | LE rank (highest to lowest) (3) | (1) - (2) | (1) - (3) |
| --- | --- | --- | --- | --- | --- | --- | --- | --- | --- |
| 1016 | *Metropolitan* | 49.4 (42.4,57.7) | 51.4 (44.1,59.9) | 89.8 (88.9,90.8) | 1 | 1 | 2 | 0 | -1 |
| 1864 | *Metropolitan* | 55.3 (50.3,60.8) | 57.7 (52.5,63.4) | 88.6 (88.0,89.3) | 2 | 4 | 4 | -2 | -2 |
| 1290 | *Metropolitan* | 58.2 (49.1,69.0) | 57.8 (48.9,68.3) | 89.2 (87.9,90.5) | 3 | 5 | 3 | -2 | 0 |
| 1270 | *Metropolitan* | 59.1 (51.9,67.4) | 57.0 (50.2,64.7) | 87.7 (86.8,88.6) | 4 | 3 | 12 | 1 | -8 |
| 2776 | *Metropolitan* | 59.5 (54.9,64.5) | 60.3 (55.6,65.4) | 87.9 (87.4,88.5) | 5 | 10 | 6 | -5 | -1 |
| 231 | *Metropolitan* | 60.2 (52.1,69.4) | 55.3 (48.2,63.5) | 87.6 (86.5,88.7) | 6 | 2 | 14 | 4 | -8 |
| 502 | *Metropolitan* | 60.3 (53.6,67.8) | 61.2 (54.5,68.7) | 88.0 (87.0,89.0) | 7 | 11 | 5 | -4 | 2 |
| 3360 | *Metropolitan* | 60.5 (54.4,67.4) | 58.5 (52.6,65.0) | 87.5 (86.8,88.2) | 8 | 7 | 17 | 1 | -9 |
| 3115 | *Metropolitan* | 60.7 (53.5,68.8) | 59.8 (52.8,67.7) | 87.6 (86.8,88.5) | 9 | 8 | 13 | 1 | -4 |
| 318 | *Metropolitan* | 60.7 (55.0,66.9) | 57.8 (52.5,63.6) | 87.7 (87.0,88.4) | 10 | 6 | 11 | 4 | -1 |
| 1298 | *Metropolitan* | 61.0 (54.3,68.6) | 60.0 (53.4,67.4) | 87.8 (87.0,88.7) | 11 | 9 | 8 | 2 | 3 |
| 311 | *Metropolitan* | 62.2 (56.9,68.0) | 62.7 (57.4,68.5) | 87.6 (86.9,88.2) | 12 | 17 | 15 | -5 | -3 |
| 1111 | *Metropolitan* | 62.8 (56.1,70.2) | 63.3 (56.6,70.8) | 86.9 (86.2,87.7) | 13 | 21 | 32 | -8 | -19 |
| 2665 | *Metropolitan* | 63.0 (55.4,71.6) | 62.8 (55.3,71.4) | 87.7 (86.7,88.6) | 14 | 18 | 10 | -4 | 4 |
| 1303 | *Metropolitan* | 63.1 (57.7,69.1) | 64.2 (58.7,70.2) | 87.4 (86.7,88.1) | 15 | 26 | 19 | -11 | -4 |
| 1480 | *Metropolitan* | 63.7 (58.3,69.5) | 63.3 (58.2,68.9) | 87.2 (86.6,87.9) | 16 | 22 | 25 | -6 | -9 |
| 1756 | *Metropolitan* | 63.8 (59.1,68.9) | 62.5 (57.9,67.5) | 87.0 (86.5,87.5) | 17 | 15 | 31 | 2 | -14 |
| 1371 | *Metropolitan* | 64.1 (58.8,69.9) | 62.8 (57.7,68.4) | 86.9 (86.3,87.4) | 18 | 19 | 35 | -1 | -17 |
| 2828 | *Metropolitan* | 64.3 (57.4,71.9) | 64.4 (57.6,72.0) | 87.2 (86.3,88.0) | 19 | 27 | 26 | -8 | -7 |
| 3214 | *Metropolitan* | 64.4 (57.5,72.3) | 63.8 (57.0,71.4) | 87.9 (87.0,88.7) | 20 | 24 | 7 | -4 | 13 |
| 1269 | *Metropolitan* | 64.7 (57.5,72.8) | 63.4 (56.7,70.9) | 87.8 (86.8,88.7) | 21 | 23 | 9 | -2 | 12 |
| 2728 | *Metropolitan* | 64.8 (56.8,74.0) | 64.8 (56.9,73.8) | 87.6 (86.5,88.6) | 22 | 28 | 16 | -6 | 6 |
| 3067 | *Metropolitan* | 65.3 (58.1,73.4) | 62.5 (55.7,70.1) | 87.4 (86.5,88.2) | 23 | 16 | 21 | 7 | 2 |
| 2232 | *Metropolitan* | 65.3 (59.0,72.4) | 63.2 (57.2,69.9) | 86.8 (86.1,87.5) | 24 | 20 | 37 | 4 | -13 |
| 2568 | *Metropolitan* | 65.8 (58.6,73.8) | 65.9 (58.8,73.8) | 86.7 (85.8,87.6) | 25 | 34 | 39 | -9 | -14 |
| 879 | *Metropolitan* | 66.2 (59.1,74.1) | 64.1 (57.5,71.5) | 86.7 (85.8,87.5) | 26 | 25 | 43 | 1 | -17 |
| 965 | *Metropolitan* | 66.2 (60.8,72.1) | 65.2 (59.9,70.9) | 87.1 (86.5,87.7) | 27 | 30 | 27 | -3 | 0 |
| 1427 | *Metropolitan* | 66.6 (61.6,72.0) | 65.2 (60.3,70.5) | 86.5 (86.0,87.1) | 28 | 31 | 50 | -3 | -22 |
| 2103 | *Metropolitan* | 66.8 (58.3,76.4) | 65.2 (57.1,74.4) | 86.7 (85.7,87.6) | 29 | 32 | 44 | -3 | -15 |
| 1871 | *Metropolitan* | 66.8 (60.8,73.3) | 61.6 (56.3,67.5) | 86.6 (86.0,87.3) | 30 | 13 | 45 | 17 | -15 |

*Notes.* CI = Confidence interval; CMF = Comparative mortality figure; LE = Life expectancy; SMR = Standardized mortality ratio.

SMR and CMF were rescaled by multiplying by 100.

Metropolitan corresponds to *dong*, urban to *eup*, and rural to *myeon*.


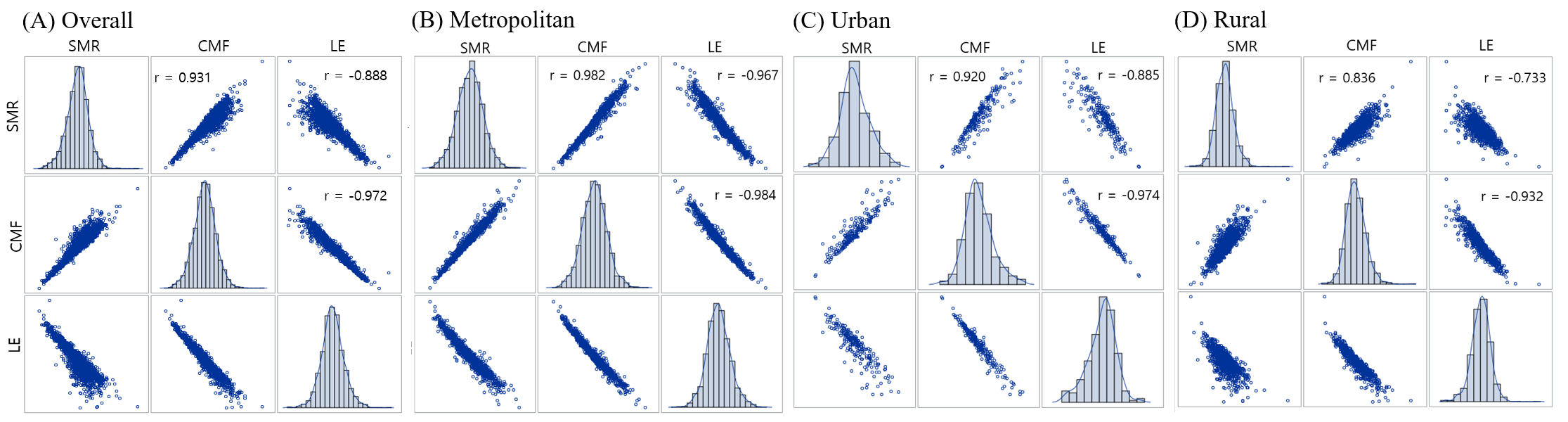


Supplementary Figure 2. Scatter plots and correlation coefficients [r] for SMR, CMF, and LE over all areas and stratified by urbanity: findings from the National Health Information Database of Korea, 2013–2017.

*Notes.* CMF = Comparative mortality figure; LE = Life expectancy; SMR = Standardized mortality ratio.

SMR and CMF were rescaled by multiplying by 100.

Metropolitan corresponds to *dong*, urban to *eup*, and rural to *myeon*.

Supplementary Table 6. Age structure, observed and expected deaths using national age-specific mortality in 2015 and age-specific mortality of the standard population, and their differences in small-areas with the minimum, maximum, and median SMR: findings from the National Health Information Database of Korea, 2013–2017

| Age group | Age-specific mortality of the SP  (1) | National age-specific mortality in 2015  (2) | (1)-(2) |  | Study population 1 | | | |  | Study population 2 | | | |  | Study population 3 | | | |
| --- | --- | --- | --- | --- | --- | --- | --- | --- | --- | --- | --- | --- | --- | --- | --- | --- | --- | --- |
|  |  |  |  |  | POP  (%) | EXP | OBS | EXP-OBS  (%) |  | POP  (%) | EXP | OBS | EXP-OBS  (%) |  | POP  (%) | EXP | OBS | EXP-OBS  (%) |
| 0 | 0.00057 | 0.00282 | -0.00225 |  | 124 (0.3) | 0.07 | 0.35 | -0.28 (-4.0) |  | 871 (3.4) | 0.50 | 2.46 | -1.95 (89.6) |  | 1016 (1.0) | 0.58 | 2.86 | -2.28 (-30.0) |
| 1-4 | 0.00014 | 0.00065 | -0.00051 |  | 548 (1.3) | 0.08 | 0.36 | -0.28 (-4.1) |  | 2319 (9.1) | 0.33 | 1.52 | -1.19 (54.6) |  | 3591 (3.4) | 0.50 | 2.35 | -1.85 (-24.3) |
| 5-9 | 0.00009 | 0.00009 | 0.00000 |  | 872 (2.1) | 0.08 | 0.08 | 0.00 (0.0) |  | 763 (3.0) | 0.07 | 0.07 | 0.00 (-0.1) |  | 3834 (3.7) | 0.35 | 0.34 | 0.01 (0.1) |
| 10-14 | 0.00009 | 0.00009 | 0.00000 |  | 1329 (3.2) | 0.13 | 0.11 | 0.01 (0.2) |  | 509 (2.0) | 0.05 | 0.04 | 0.00 (-0.2) |  | 4229 (4.0) | 0.40 | 0.36 | 0.03 (0.5) |
| 15-19 | 0.00025 | 0.00021 | 0.00004 |  | 2122 (5.1) | 0.53 | 0.44 | 0.09 (1.3) |  | 1112 (4.4) | 0.28 | 0.23 | 0.05 (-2.1) |  | 5836 (5.6) | 1.46 | 1.22 | 0.25 (3.2) |
| 20-24 | 0.00034 | 0.00033 | 0.00001 |  | 2628 (6.4) | 0.89 | 0.86 | 0.03 (0.4) |  | 2033 (8.0) | 0.69 | 0.67 | 0.02 (-1.0) |  | 7617 (7.3) | 2.59 | 2.51 | 0.08 (1.1) |
| 25-29 | 0.00046 | 0.00047 | -0.00001 |  | 2356 (5.7) | 1.09 | 1.11 | -0.02 (-0.3) |  | 3477 (13.6) | 1.61 | 1.64 | -0.03 (1.5) |  | 7795 (7.4) | 3.61 | 3.68 | -0.07 (-1.0) |
| 30-34 | 0.00062 | 0.00061 | 0.00001 |  | 2521 (6.1) | 1.57 | 1.54 | 0.03 (0.5) |  | 4963 (19.5) | 3.09 | 3.03 | 0.06 (-2.8) |  | 9756 (9.3) | 6.07 | 5.95 | 0.12 (1.6) |
| 35-39 | 0.00085 | 0.00080 | 0.00005 |  | 2433 (5.9) | 2.07 | 1.95 | 0.12 (1.8) |  | 2718 (10.7) | 2.31 | 2.17 | 0.14 (-6.3) |  | 8352 (8.0) | 7.10 | 6.68 | 0.42 (5.5) |
| 40-44 | 0.00129 | 0.00126 | 0.00003 |  | 3014 (7.3) | 3.88 | 3.78 | 0.10 (1.5) |  | 1454 (5.7) | 1.87 | 1.83 | 0.05 (-2.2) |  | 7918 (7.6) | 10.20 | 9.94 | 0.26 (3.5) |
| 45-49 | 0.00200 | 0.00197 | 0.00003 |  | 3588 (8.7) | 7.19 | 7.08 | 0.12 (1.7) |  | 1386 (5.4) | 2.78 | 2.73 | 0.05 (-2.1) |  | 8096 (7.7) | 16.23 | 15.97 | 0.27 (3.5) |
| 50-54 | 0.00305 | 0.00296 | 0.00009 |  | 4208 (10.2) | 12.81 | 12.44 | 0.38 (5.5) |  | 1588 (6.2) | 4.84 | 4.69 | 0.14 (-6.5) |  | 9305 (8.9) | 28.33 | 27.50 | 0.83 (11.0) |
| 55-59 | 0.00428 | 0.00416 | 0.00012 |  | 4352 (10.5) | 18.64 | 18.10 | 0.55 (8.0) |  | 1079 (4.2) | 4.62 | 4.49 | 0.14 (-6.2) |  | 8953 (8.5) | 38.35 | 37.23 | 1.13 (14.8) |
| 60-64 | 0.00631 | 0.00615 | 0.00016 |  | 3565 (8.6) | 22.51 | 21.92 | 0.59 (8.5) |  | 569 (2.2) | 3.59 | 3.50 | 0.09 (-4.3) |  | 6375 (6.1) | 40.25 | 39.20 | 1.05 (13.8) |
| 65-69 | 0.00989 | 0.00943 | 0.00046 |  | 2772 (6.7) | 27.43 | 26.15 | 1.28 (18.6) |  | 275 (1.1) | 2.72 | 2.59 | 0.13 (-5.8) |  | 4634 (4.4) | 45.85 | 43.72 | 2.13 (28.1) |
| 70-74 | 0.01789 | 0.01710 | 0.00079 |  | 2256 (5.5) | 40.35 | 38.59 | 1.77 (25.7) |  | 143 (0.6) | 2.56 | 2.45 | 0.11 (-5.1) |  | 3435 (3.3) | 61.44 | 58.75 | 2.69 (35.4) |
| 75-79 | 0.03260 | 0.03152 | 0.00108 |  | 1542 (3.7) | 50.27 | 48.60 | 1.66 (24.2) |  | 87 (0.3) | 2.84 | 2.74 | 0.09 (-4.3) |  | 2269 (2.2) | 73.96 | 71.52 | 2.45 (32.2) |
| 80-84 | 0.05894 | 0.05670 | 0.00224 |  | 800 (1.9) | 47.15 | 45.36 | 1.79 (26.1) |  | 71 (0.3) | 4.18 | 4.03 | 0.16 (-7.3) |  | 1068 (1.0) | 62.94 | 60.55 | 2.39 (31.5) |
| 85+ | 0.12728 | 0.13056 | -0.00328 |  | 322 (0.8) | 40.98 | 42.04 | -1.06 (-15.4) |  | 71 (0.3) | 9.04 | 9.27 | -0.23 (10.7) |  | 707 (0.7) | 89.99 | 92.30 | -2.32 (-30.5) |
| Total |  |  |  |  | 41352 (100.0) | 277.73 | 270.85 | 6.87 (100.0) |  | 25488 (100.0) | 47.96 | 50.14 | -2.18 (100.0) |  | 104786 (100.0) | 490.23 | 482.63 | 7.59 (100.0) |
| SMR |  |  |  |  | $100\cdot\left( 1-\frac{6.87}{277.73} \right)=$ 97.5 | | | |  | $100\cdot\left\{ 1-(\frac{-2.18}{47.96}) \right\}=$104.6 | | | |  | $100\cdot\left( 1-\frac{7.59}{490.23} \right)=$98.5 | | | |

Notes. EXP = Expected number of deaths; MR = Mortality ratio; OBS = number of observed deaths; POP = Population; SMR = Standardized mortality ratio; SP = Standard population.

SMR and CMF were rescaled by multiplying by 100.
